# Cervical sagittal alignment after Prestige LP cervical disc replacement: radiological results and clinical impacts from a single-center experience

**DOI:** 10.1186/s12891-021-03962-x

**Published:** 2021-01-15

**Authors:** Xiaofei Wang, Yang Meng, Hao Liu, Hua Chen, Beiyu Wang, Ying Hong

**Affiliations:** 1 Department of Orthopaedic Surgery, West China Hospital, Sichuan University, No. 37 Guo Xue Xiang, Chengdu, 610041 Sichuan China; 2 Department of Anesthesia and Operation Center/West China School of Nursing, West China Hospital, Sichuan University, No. 37 Guo Xue Xiang, Chengdu, 610041 Sichuan China

**Keywords:** Cervical disc replacement, Cervical sagittal alignment, T1 slope, Sagittal vertical axis, Heterotopic ossification, Adjacent segment degeneration

## Abstract

**Background:**

Cervical disc replacement (CDR) has been widely used to treat one- and two-level cervical degenerative disc disease. Studies have shown the effectiveness of CDR in preserving range of motion (ROM) and delaying adjacent segment degeneration (ASD). Cervical sagittal alignment is an important factor affecting favorable clinical outcomes in cervical spine surgery. This study aimed to explore whether cervical sagittal alignment can be maintained after CDR and to identify the impact of cervical sagittal alignment on outcomes after CDR.

**Methods:**

This was a single-center, retrospective study. 132 patients who underwent one-level CDR were included. Cervical sagittal alignments, including cervical lordosis (CL), segmental alignment (SA), sagittal vertical axis (SVA), T1 slope (T1s), and T1s minus CL (T1s-CL), were measured. The effects of cervical sagittal alignment on the CDR outcomes were analyzed. Patients were divided into the heterotopic ossification (HO) group and ASD group to determine the potential impacts of cervical sagittal parameters.

**Results:**

The cervical sagittal alignment parameters, except for the SVA, were significantly improved after CDR and showed decreasing trends at the last follow-up. Significantly higher CL and T1s were found in patients with better ROM after CDR. SVA ≥ 20 mm increased the risk of anterior HO (odds ratio = 2.945, *P* = 0.007). Significantly kyphotic SA and lower T1s values were found in the ASD patients than in the non-ASD patients (*P* < 0.05). Patients with ASD at the inferior level showed significantly worse CL (*P* < 0.05).

**Conclusion:**

CDR had limited function of improving cervical sagittal alignment. Poor cervical sagittal alignment after CDR was associated with HO, ASD, and less ROM.

**Supplementary Information:**

The online version contains supplementary material available at 10.1186/s12891-021-03962-x.

## Background

Cervical sagittal balance plays an important role in transferring axial loads and maintaining the mechanical functions of the cervical spine. An imbalance in cervical sagittal alignment may lead to neck pain and accelerate the degeneration of the cervical spine [1, 2]. Previous studies have proven that after anterior cervical discectomy and fusion (ACDF), abnormal cervical sagittal alignment can affect patients’ quality of life [1, 3, 4]. In addition, segmental kyphosis of the cervical spine increases the risk of adjacent segment degeneration (ASD) after ACDF [5]. However, whether the same outcomes occur after cervical disc replacement (CDR) remains unknown.

CDR has been recently proposed as a method of preserving the mobility of the cervical spine [6]. This technique aims to restore the physiological function of the cervical spine to the greatest extent possible. Therefore, maintaining cervical sagittal balance after CDR is of great importance. However, most studies have only focused on the cervical curvature after CDR, and other important cervical sagittal alignment parameters, including the sagittal vertical axis (SVA), T1-slope (T1s), and T1-slope minus cervical lordosis (T1s-CL), have rarely been investigated [7]. Whether CDR can restore normal sagittal balance has been poorly studied. Moreover, the impact of cervical sagittal alignment on the outcomes and complications after CDR has been poorly studied.

In the current study, cervical sagittal alignment parameters were evaluated in patients who underwent CDR with a minimum follow-up of 24 months. By reviewing the radiological and clinical data, we aimed to answer the following questions: 1) Can CDR restore the cervical sagittal balance after surgery? 2) Will cervical sagittal alignment affect the outcomes after CDR? We conducted this study to gain a better understanding of the importance of cervical sagittal balance after CDR.

## Methods

### Study design

This was an observational, single-center study. Patients who underwent single-level cervical disc replacement using the PRESTIGE LP (Medtronic Sofamor Danek, Memphis, Tennessee, USA) from 2008 to 2017 were included in this study. All surgeries were performed by the same senior surgeon with the same surgical technique. The inclusion criteria were as follows: 1) 18–65 years old; 2) single-level cervical degenerative disc disease causing symptomatic radiculopathy or myelopathy between C3 and C7; and 3) failure of conservative treatment for at least 12 weeks. The exclusion criteria were as follows: 1) instability, irreducible kyphosis, or severe degeneration at the index level; and 2) prior cervical spine surgery. In total, 132 patients with at least 24 months follow-up were enrolled in this study.

### Radiological evaluation

**Fig. 1:**
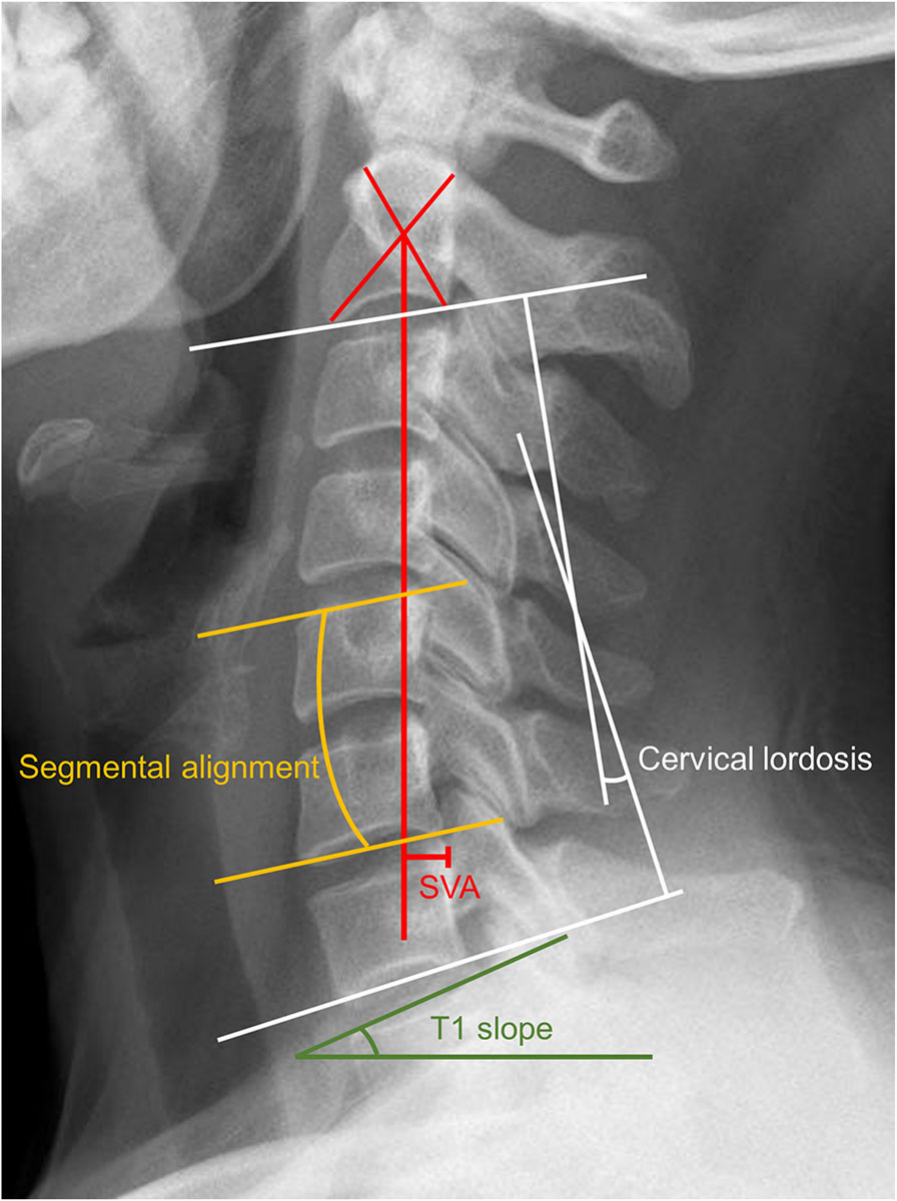
Radiological evaluation of the cervical sagittal alignment parameters. (1) White line: Cervical lordosis (CL), the angle between a line parallel to the inferior endplate of C2 and a line parallel to the inferior endplate of C7. (2) Yellow line: Segment alignment (SA), the angle between the lines parallel to the superior and inferior aspect of the surgical segmental vertebral bodies. (3) Red line: Sagittal vertical axis (SVA), the distance between a plumb line from the center of C2 and the superior posterior corner of C7; (4) Green line: T1-slope (T1s), the angle between a line parallel to the superior endplate of T1 and a horizontal line

Cervical sagittal alignment parameters were measured on the lateral radiographs, and range of motion (ROM) was measured on the flexion-extension radiographs [8–10]. Lateral radiographs of the cervical spine were obtained in a standing position, and the patients were asked to look straight ahead, with their hips and knees extended [11]. The patients were also asked to lean their back against the fluorescent screen. The following cervical sagittal alignment parameters were evaluated as previously described [11](Fig. 1): 1) Cervical lordosis (CL); 2) Segment alignment (SA); 3) SVA; 4) T1s; and 5) T1s-CL.

### Clinical outcomes

The Japanese Orthopaedic Association (JOA) score, neck disability index (NDI) score, and visual analog scale (VAS) score were recorded to evaluate the clinical symptoms of the patients. According to the position of HO on the lateral radiographs, the patients were divided into the anterior heterotopic ossification (AHO) group and posterior heterotopic ossification (PHO) group [12]. ASD was considered to exist when one of the following criteria were met [5, 13]: 1) new or enlarging osteophyte formation at the anterior or posterior border of vertebrae; 2) narrowing of the intervertebral disc space > 25%; 3) ossification of the anterior or posterior longitudinal ligament.

### Statistical analysis

All measurements were performed by two surgeons independently. Normality was tested by the Kolmogorov-Smirnov test (sample size > 50) or the Shapiro-Wilk test (sample size < 50). For the continuous variables, the mean value was calculated, and the data are presented as the mean ± standard deviation (SD). For the categorical variables, discrepancies were settled by consultation with a senior surgeon until a consensus was reached. The paired t-test (for normally distributed data) or Wilcoxon signed rank test (for nonnormally distributed data) was used to compare the changes in the sagittal alignment parameters among the follow-up times. Student’s t-test (for normally distributed data) or the Mann-Whitney U test (for nonnormally distributed data) was used to compare the data between the two groups. The chi-square test was used to analyze categorical variables. The Pearson or Spearman correlation coefficient was calculated to assess the correlation between two continuous variables. All statistical analyses were performed using SPSS software, version 23.0 (SPSS Inc., Chicago, IL). A two-tailed *p*-value of < 0.05 was considered statistically significant.

## Results

### Demographic data

In total, 132 patients were included in the current study (Table 1). The mean age of the patients was 43.6 ± 7.6 years. The median follow-up time was 33.5 months (25% quartile: 24.0, 75% quartile: 56.8; range 24–120 months). The symptoms of the patients were relieved significantly after surgery.

**Table 1:** General Information of Patients

Variable	Value
Population (number of cases)	132
Average age (years)	43.6 ± 7.6
Gender (Female/Male, number of cases)	66/66
Body mass index (kg/m^2^)	22.4 ± 2.7
Surgical level (number of cases)	
C4/5	10
C5/6	114
C6/7	8
Average follow-up (months)	43.2 ± 22.6
Preoperative JOA score	12.2 ± 1.9
Last Follow-up JOA score	16.0 ± 0.8^a^
Preoperative NDI score	22.7 ± 4.8
Last Follow-up NDI score	5.7 ± 0.8^a^
Preoperative VAS score	5.6 ± 1.3
Last Follow-up VAS score	1.6 ± 0.5^a^

*JOA* Japanese Orthopaedic Association, *NDI* neck disability index, *VAS* visual analog scale

^a^Significant difference compared with preoperative values

### Can CDR restore the cervical sagittal balance?

Table 2 demonstrates the cervical sagittal alignment parameters at different follow-up times. SVA did not change significantly before and after the operation. However, the other four parameters (CL, SA, T1s, T1s-CL) changed significantly after CDR. Except for SVA, the parameters showed decreasing trends during the course of this study. The values of CL and SA at the last follow-up were significantly improved compared with the preoperative values.

**Table 2:** Effects of Cervical Disc Replacement on Cervical Sagittal Alignment Parameters

Variable	Preoperative	Postoperative	Last Follow-up	*P* value (Pre-post)	*P*^*†*^ value (Post-last)	*P*^*#*^ value (Pre-last)
CL (°)	8.9 ± 10.3	15.4 ± 9.0	12.5 ± 8.3	**< 0.001**	**< 0.001**	**< 0.001**
SA (°)	−3.3 ± 5.6	1.2 ± 4.8	−2.4 ± 5.0	**< 0.001**	**< 0.001**	**0.047**
SVA (mm)	16.6 ± 8.6	16.9 ± 7.7	16.7 ± 7.1	0.627	0.774	0.885
T1s (°)	24.8 ± 6.5	27.6 ± 6.8	22.3 ± 10.5	**< 0.001**	**< 0.001**	**0.113**
T1s-CL (°)	15.8 ± 8.6	12.2 ± 8.0	9.8 ± 12.2	**< 0.001**	**0.256**	**< 0.001**

Postoperative, within 1 week after surgery; *P* value, comparison between preoperative and postoperative values; *P*^***†***^ value, comparison between postoperative and the last follow-up values; *P*^*#*^ value, comparison between preoperative and the last follow-up values

*CL* C2–7 lordosis, *SA* segmental angle, *SVA* sagittal vertical axis, *T1s* T1 slope, *T1s-CL* T1 slope minus C2–7 lordosis

### Will cervical sagittal alignment affect the outcomes after CDR?

Significant positive correlations were noticed between CL and the last segmental ROM, and similar results were noticed in postoperative SA and postoperative T1s. In addition, the last T1s-CL was negatively correlated with the last segmental ROM (Supplementary Table 1). Patients were divided into the less mobile group (ROM < 5°) or more mobile group (ROM ≥5°) according to the ROM of the artificial disc at the last follow-up (Table 3) [14]. Both postoperative CL and CL at the last follow-up were significantly more lordotic in the more mobile group (*P* < 0.01 for each value). In addition, the more mobile group had significantly higher T1s after surgery (*P* < 0.05). At the last follow-up, the less mobile group was noticed with significantly larger SVA and higher T1s-CL (*P* < 0.05 for each value).

**Table 3:** Effects of Sagittal Alignment Parameters on Range of Motion After Cervical Disc Replacement

Variable	Less mobile disc (*n* = 30)	More mobile disc (*n* = 102)	*P* value
**Postoperative**			
CL (°)	11.5 ± 9.8	16.6 ± 8.4	**0.014**^**a**^
SA (°)	−0.5 ± 4.6	1.7 ± 4.7	0.065
SVA (mm)	17.7 ± 9.3	16.7 ± 7.2	0.939
T1s (°)	25.1 ± 6.8	28.3 ± 6.7	**0.026**^**a**^
T1s-CL (°)	12.0 ± 6.1	12.2 ± 8.5	0.515
**Last Follow-up**			
CL (°)	8.4 ± 7.8	13.6 ± 8.1	**0.002**^**a**^
SA (°)	−3.2 ± 4.9	−2.2 ± 5.0	0.364
SVA (mm)	19.3 ± 7.8	15.9 ± 6.8	**0.039**^**a**^
T1s (°)	23.2 ± 9.6	22.0 ± 10.8	0.680
T1s-CL (°)	14.1 ± 8.5	8.5 ± 12.8	**0.018**^**a**^

*CL* C2–7 lordosis, *SA* segmental angle, *SVA* sagittal vertical axis, *T1s* T1 slope, *T1s-CL* T1 slope minus C2–7 lordosis, *ROM* range of motion

^a^Significant difference between two groups

Cervical sagittal alignment affected the heterotopic bone formation after CDR (Table 4). The postoperative SVA in the AHO group was significantly larger than that in the non-AHO group (20.4 ± 7.7 mm vs. 15.5 ± 7.3 mm, *P* = 0.003). Similarly, the SVA at the last follow-up was also significantly larger in the AHO group than in the non-AHO group (20.3 ± 8.5 mm vs. 15.2 ± 5.9 mm, *P* = 0.001). In addition, the postoperative T1s-CL was significantly higher in the AHO group than in the non-AHO group (14.5 ± 6.5° vs. 11.2 ± 8.4°, *P* = 0.014). We divided patients in to SVA ≥ 20 mm group (*n* = 40) or SVA < 20 mm group (*n* = 92) according to the postoperative value [15]. The AHO rate was significantly higher in the SVA ≥ 20 mm group (45.0% vs. 21.7%, *P* = 0.007, OR = 2.945, 95% CI = 1.329, 6.528, data not shown).

**Table 4:** Effects of Sagittal Alignment Parameters on Heterotopic Ossification After Cervical Disc Replacement

Variable	Non-AHO (*n* = 94)	AHO (*n* = 38)	*P* value	Non-PHO (*n* = 82)	PHO (*n* = 50)	*P* value
**Postoperative**						
CL (°)	16.2 ± 8.8	13.3 ± 9.1	0.087	15.2 ± 8.9	15.7 ± 9.1	0.778
SA (°)	1.3 ± 5.4	0.7 ± 2.9	0.310	0.8 ± 4.4	1.8 ± 5.4	0.255
SVA (mm)	15.5 ± 7.3	20.4 ± 7.7	**0.003**^**a**^	17.3 ± 7.6	16.2 ± 7.9	0.368
T1s (°)	27.5 ± 7.3	27.8 ± 5.7	0.710	27.6 ± 6.8	27.5 ± 6.9	0.659
T1s-CL (°)	11.2 ± 8.4	14.5 ± 6.5	**0.014**^**a**^	12.4 ± 8.0	11.8 ± 8.2	0.573
**Last Follow-up**						
CL (°)	12.9 ± 8.1	11.4 ± 8.6	0.439	12.4 ± 8.3	12.6 ± 8.3	0.881
SA (°)	−2.3 ± 5.3	−2.8 ± 4.1	0.615	−3.2 ± 4.8	−1.1 ± 5.1	**0.020**^**a**^
SVA (mm)	15.2 ± 5.9	20.3 ± 8.5	**0.001**^**a**^	17.6 ± 8.0	15.1 ± 5.0	**0.032**^**a**^
T1s (°)	23.0 ± 9.8	20.4 ± 11.9	0.415	22.8 ± 11.1	21.5 ± 9.5	0.458
T1s-CL (°)	10.1 ± 11.7	9.0 ± 13.3	0.833	10.4 ± 11.2	8.9 ± 13.7	0.940

*CL* C2–7 lordosis, *SA* segmental angle, *SVA* sagittal vertical axis, *T1s* T1 slope, *T1s-CL* T1 slope minus C2–7 lordosis, *NDI* neck disability index, *AHO* anterior heterotopic ossification, *PHO* posterior heterotopic ossification

^a^Significant difference between two groups

Adjacent segment degeneration at the superior (SASD) or inferior level (IASD) was also affected by cervical sagittal alignments (Table 5). Patients with ASD showed significant kyphotic SA and smaller T1s postoperatively compared with the non-ASD patients (*P* < 0.05 for each value). At the last follow-up, both CL and T1s were significantly smaller in the ASD group (*P* < 0.05 for each value), and SA in the ASD group was more kyphotic (*P* < 0.05 in the IASD group). In addition, for the IASD group, the postoperative CL (11.9 ± 10.7° vs. 16.3 ± 8.2°, *P* = 0.048) was significantly smaller than those in the non-IASD group.

**Table 5:** Effects of Sagittal Alignment Parameters on Adjacent Segment Degeneration After Cervical Disc Replacement

Variable	Non-SASD (*n* = 114)	SASD (*n* = 18)	*P* value	Non-IASD (*n* = 104)	IASD (*n* = 28)	*P* value
**Postoperative**						
CL (°)	15.9 ± 8.3	12.4 ± 12.4	0.189	16.3 ± 8.2	11.9 ± 10.7	**0.048**^**a**^
SA (°)	1.7 ± 4.5	−2.1 ± 5.4	**0.006**^**a**^	1.8 ± 4.9	−1.2 ± 3.7	**0.001**^**a**^
SVA (mm)	17.0 ± 8.1	16.3 ± 4.8	0.740	16.7 ± 8.1	17.5 ± 6.1	0.609
T1s (°)	28.0 ± 7.1	25.2 ± 3.9	**0.020**^**a**^	28.2 ± 7.1	25.1 ± 5.4	**0.034**^**a**^
T1s-CL (°)	12.1 ± 7.4	12.8 ± 11.5	0.740	11.9 ± 8.0	13.3 ± 8.2	0.476
**Last Follow-up**						
CL (°)	13.1 ± 8.0	8.4 ± 9.3	**0.026**^**a**^	13.3 ± 7.8	9.2 ± 9.2	**0.018**^**a**^
SA (°)	−2.2 ± 4.9	−4.0 ± 5.7	0.160	−1.8 ± 5.1	−4.8 ± 3.8	**0.001**^**a**^
SVA (mm)	16.9 ± 7.2	15.3 ± 6.5	0.482	16.5 ± 6.6	17.3 ± 8.9	0.665
T1s (°)	23.2 ± 10.3	16.4 ± 10.1	**0.004**^**a**^	24.0 ± 9.5	15.7 ± 11.5	**< 0.001**^**a**^
T1s-CL (°)	10.1 ± 11.6	8.0 ± 15.4	0.926	10.7 ± 11.5	6.5 ± 14.1	0.212

*CL* C2–7 lordosis, *SA* segmental angle, *SVA* sagittal vertical axis, *T1s* T1 slope, *T1s-CL* T1 slope minus C2–7 lordosis, *NDI* neck disability index, *SASD* superior adjacent segment degeneration, *IASD* inferior adjacent segment degeneration

^a^Significant difference between two groups

At the last follow-up, the JOA, NDI, and VAS values were not correlated with the cervical sagittal alignment parameters (Supplementary Table 2). In addition, we did not observe a correlation between the clinical outcomes and changes in the cervical sagittal parameters (*P* > 0.05).

## Discussion

CDR has been introduced to preserve the ROM at the surgical level and to delay the degeneration process at adjacent levels. Although a large number of studies have focused on the clinical outcomes and complications of CDR, the relationship between CDR and cervical sagittal alignment has rarely been studied. Normal sagittal alignment in the cervical spine plays important roles in attaining global spinal balance, transferring axial loads, and maintaining mobility [1, 16]. In cervical arthrodesis, a change in SVA or T1s-CL has been shown to be related to worse clinical outcomes, as measured by the NDI score [3, 4], and kyphotic SA has been shown to be a risk factor for ASD [5]; in addition, T1s has been shown to be a predictive factor for subsidence and pseudarthrosis [16]. However, whether these cervical sagittal parameters affect CDR is currently unknown. We performed this retrospective study to answer the following questions: 1) Can CDR restore the cervical sagittal balance after surgery? 2) Will the relationships between cervical sagittal alignments change after CDR? 3) Will cervical sagittal alignment affect the outcomes after CDR?

CDR cannot restore the cervical sagittal alignment. In our study, the overall and segmental alignment after CDR improved significantly. However, consistent with previous studies, a loss of global and segmental lordosis was observed at the last follow-up [9, 17, 18]. Notwithstanding, both CL and SA at the last follow-up were significantly no worse than those before surgery. In addition, an increase in T1s after CDR was found, while the T1s at the last follow-up return to the preoperative level. These results reveal that the function of CDR in restoring cervical sagittal alignment is poor.

In our study, more lordotic cervical alignment and higher T1s were noticed in the more mobile group, which indicates that the motion function of the artificial disc can be affected by cervical sagittal alignments. Cervical sagittal alignments may affect the ROM indirectly through the shell angle of artificial discs. For instance, Rabin et al. found a negative correlation between the shell angle and the ROM of Prodisc-C artificial disc (Synthes Inc., West Chester, Pennsylvania, USA) [19]. Similarly, we found that the postoperative shell angle in the more mobile group was significantly better than the less mobile group (5.8 ± 4.8° vs. 2.6 ± 6.9°, *P* = 0.022, data not shown). Therefore, sagittal alignment reconstruction has important implications for the basic function (motion function) of CDR.

The relationship between HO and cervical sagittal alignment after CDR has not yet been studied. In the current study, we found that patients with postoperative SVA ≥ 20 mm increased the risk of AHO about 2-fold. A higher SVA may increase the stress in the anterior region of the cervical vertebrae and stimulate bone formation, which appears as HO after CDR. In addition, we think that the differences in SA and SVA at the last follow-up between the PHO and the non-PHO patients were effects rather than causes of PHO.

Segmental kyphosis has been considered a risk factor for ASD in patients who undergo ACDF [5]. Lee et al. reviewed 12 patients who underwent CDR and found that non-ASD patients had a more lordotic SA postoperatively than ASD patients [20]. In this study, we found that both the SASD group and the IASD group showed a significantly more kyphotic SA after surgery, which indicated that postoperative segmental kyphosis is a potential risk factor for ASD after CDR. In addition to more severe kyphotic SA, smaller CL was also found to potentially lead to ASD in the current study. The load transmission pattern in the cervical spine can explain the relationship between an abnormal cervical curvature and ASD. Physiologically, a normal cervical curvature allows the cervical spine to distribute 36% of the axial load through the anterior column and 64% through the posterior column [21]. However, both segmental kyphosis and reduced cervical lordosis tend to shift the axial load from the posterior column to the anterior column. This shift can potentially increase the mechanical stress at adjacent levels. The increased stress may interfere with the nutrition supply of intervertebral discs and accelerate the degeneration process, which may eventually lead to ASD [1]. In addition, a lower T1s was found to be associated with ASD. Similarly, a recent study in the lumbar pelvic region showed that a higher PI (an equivalent parameter to T1s) was related to a higher rate of disc degeneration [22]. However, our results were inconsistent with that reported by Shen et al., who found that patients with a higher T1s showed a higher ASD rate [23]. Theoretically, T1s increases to compensate for a lordotic cervical curvature or decreases to compensate for a straight cervical curvature so that the individual can maintain a horizontal gaze. In this regard, ASD should be related to a lower T1s. However, further studies are needed to confirm this relationship.

In the current study, we did not find that these cervical sagittal parameters had any impact on the patient-reported outcomes after CDR, which was consistent with the results reported by Yang et al. [24] However, Guerin et al. [25] reviewed the data of 40 Mobi-C CDR patients and found significant positive correlations between postoperative SA and the postoperative SF-36 score (*r =* 0.349, *P* = 0.027) and between the postoperative shell angle and postoperative VAS neck pain score (*r =* 0.422, *P* = 0.007). Shen et al. [23] measured the T1s in 90 patients who underwent one-level Bryan CDR and found that a higher T1s was related to more obvious neck pain at the 6-year follow-up. We think that the small sample size and retrospective design might yield a statistical power that is insufficiently high to detect the actual relationships between cervical sagittal alignment parameters and patient-reported outcomes. In addition, both the VAS neck pain score and NDI score are subjective indicators that might be influenced by subjective factors, such as the researchers’ intentions and their investigative methods [26, 27].

In conclusion, our study suggested the importance of normal cervical sagittal alignment after CDR. CDR had limited function of improving cervical sagittal alignment. Besides, poor cervical sagittal alignment after CDR was associated with HO, ASD, and less ROM. Since CDR shows poor ability to restore the segmental alignment, there is a greater risk of developing ASD. Therefore, whether CDR can reduce the ASD rate remains further confirmation. In addition, patients who undergo CDR should pay more attention to their neck muscle strength and lifestyle to achieve better cervical sagittal alignment.

A major limitation in this study was that we included patients with a minimum follow-up of 2 years, while the incidence of HO and ASD may be more accurate in a study with a longer follow-up period. However, our study has shown that postoperative complications are related to poor cervical sagittal alignment. Therefore, maintaining normal sagittal balance is important after CDR. Another limitation is that we did not calculate cut-off values to predict the occurrence of complications. Future studies with logistic regression analysis are needed to confirm the values of the cervical sagittal parameters useful for predicting complications after CDR. Besides, this study did not focus on the ROM-limiting HO, which has more important clinical significance than the whole HO. Unfortunately, the number of patients with ROM-limiting HO was small, therefore, the small sample size did not allow us to reach a high statistical power to detect the difference between groups. Further studies with longer follow-up time are needed to focus on the association of high-grade HO with clinical and radiological outcomes. In addition, our results of this single-center, retrospective study should be interpreted with caution because of heterogeneous follow-up time points, lack of multicenter verification, and categorizing continuous variables.

## Conclusion

CDR had limited function of improving cervical sagittal alignment. Poor cervical sagittal alignment after CDR was associated with HO, ASD, and less ROM.

## Supplementary Information

**Additional file 1: Supplementary Table 1**. Correlation Between Cervical Sagittal Alignment Parameters and Segmental Range of Motion (At the Last Follow-up) After Cervical Disc Replacement.

**Additional file 2: Supplementary Table 2.** Correlation Between Cervical Sagittal Alignment Parameters and Patient Reported Outcomes (At the Last Follow-up) After Cervical Disc Replacement.

## Data Availability

Datasets are available from the corresponding author on a reasonable request.

## Abbreviations

ACDF: Anterior Cervical Discectomy and Fusion
ASD: Adjacent Segment Degeneration
CDR: Cervical Disc Replacement
CL: Cervical Lordosis
HO: Heterotopic Ossification
JOA: Japanese Orthopaedic Ossiciation
NDI: Neck Disability Index
OR: Odds Ratio
ROM: Range of Motion
SD: Standard Deviation
SVA: Sagittal Vertical Axis
T1s: T1 Slope
T1s-CL: T1 Slope Minus Cervical Lordosis
VAS: Visual Analog Scale
