# Supplementary material for: Cervical sagittal alignment after Prestige LP cervical disc replacement: radiological results and clinical impacts from a single-center experience
Source: BMC Musculoskelet Disord. 2021 Jan 15;22:82. doi: 10.1186/s12891-021-03962-x (PMC7809768; doi:10.1186/s12891-021-03962-x)
Supplement: Supplementary file 1 — Additional file 1: Supplementary Table 1. Correlation Between Cervical Sagittal Alignment Parameters and Segmental Range of Motion (At the Last Follow-up) After Cervical Disc Replacement. [file 12891_2021_3962_MOESM1_ESM.docx]

**Supplementary Table 1. Correlation Between Cervical Sagittal Alignment Parameters and Segmental Range of Motion (At the Last Follow-up) After Cervical Disc Replacement.**

| Variable | CL | SA | SVA | T1s | T1s-CL |
| --- | --- | --- | --- | --- | --- |
| **Postoperative** | 0.286** | 0.292** | -0.023 | 0.269** | -0.033 |
| **Last** **Follow-up** | 0.304** | 0.162 | -0.069 | 0.061 | -0.218* |

CL, C2-7 lordosis; SA, segmental angle; SVA, sagittal vertical axis; T1s, T1 slope; T1s-CL, T1 slope minus C2-7 lordosis; ROM, range of motion.

***** indicates a *P* value < 0.05; ****** indicates a *P* value < 0.01.
