# Supplementary material for: Cervical sagittal alignment after Prestige LP cervical disc replacement: radiological results and clinical impacts from a single-center experience
Source: BMC Musculoskelet Disord. 2021 Jan 15;22:82. doi: 10.1186/s12891-021-03962-x (PMC7809768; doi:10.1186/s12891-021-03962-x)
Supplement: Supplementary file 2 — Additional file 2: Supplementary Table 2. Correlation Between Cervical Sagittal Alignment Parameters and Patient Reported Outcomes (At the Last Follow-up) After Cervical Disc Replacement. [file 12891_2021_3962_MOESM2_ESM.docx]

**Supplementary Table 2. Correlation Between Cervical Sagittal Alignment Parameters and Patient Reported Outcomes (At the Last Follow-up) After Cervical Disc Replacement.**

| Variable | CL | SA | SVA | T1s | T1s-CL |
| --- | --- | --- | --- | --- | --- |
| **JOA score** |  |  |  |  |  |
| Correlation | 0.023 | 0.136 | 0.130 | -0.009 | 0.063 |
| P value | 0.797 | 0.120 | 0.137 | 0.920 | 0.474 |
| **NDI score** |  |  |  |  |  |
| Correlation | -0.146 | -0.105 | -0.031 | -0.016 | 0.101 |
| P value | 0.094 | 0.231 | 0.727 | 0.858 | 0.248 |
| **VAS score** |  |  |  |  |  |
| Correlation | 0.086 | -0.106 | 0.155 | -0.058 | -0.172 |
| P value | 0.327 | 0.226 | 0.076 | 0.506 | 0.154 |

CL, C2-7 lordosis; SA, segmental angle; SVA, sagittal vertical axis; T1s, T1 slope; T1s-CL, T1 slope minus C2-7 lordosis; ROM, range of motion; JOA, Japanese Orthopaedic Association; NDI, Neck disability index; VAS, Visual analog scale.

***** indicates significant difference.
